# CircRNA regulates the liquid-liquid phase separation of ATG4B, a novel strategy to inhibit cancer metastasis?

**DOI:** 10.15698/cst2024.05.296

**Published:** 2024-05-24

**Authors:** Ziyuan Guo, Yang Chen, Yaran Wu, Jiqin Lian

**Affiliations:** 1 Department of Clinical Biochemistry, Faculty of Pharmacy and Laboratory Medicine, Army Medical University, Chongqing, China.

**Keywords:** autophagy, ATG4B, anoikis, liquid-liquid phase separation, circSPECC1, lopinavir

## Abstract

Anoikis is a common programmed death for most of detached cells, but cancer cells can obtain anoikis resistance to facilitate their distant metastasis through the circulation system. Researches have indicated that enhanced autophagic flux accounts for the survival of many cancer cells under detached conditions. Targeting ATG4B, the key factor of autophagy progress, can inhibit cancer metastasis *in vitro*, but ATG4B-deficient mice are susceptible to many serious diseases, which indicates the potential uncontrolled side effects of direct targeting of ATG4B. In our recent research, we confirmed that ATG4B is a novel RNA binding protein in the gastric cancer (GC) cell. It interacts with *circ*SPECC1 which consequently facilitates the liquid-liquid phase separation and ubiquitination of ATG4B. Additionally, the m^6^A reader ELAVL1 inhibits the expression of *circ*SPECC1 to enhance the expression of ATG4B and anoikis resistance of GC cells. Further, we screened out an FDA-approved compound, lopinavir, to restore *circ*SPECC1 abundance and suppress GC metastasis. In conclusion, our research identified a novel signal pathway (ELAVL1-*circ*SPECC1-ATG4B-autophagy) to facilitate anoikis resistance and metastasis of GC cells and screened out a compound with clinical application potential to block this pathway, providing a novel strategy for the prevention of GC metastasis.

Metastasis is the leading cause of death in cancer patients. Currently, there are no effective treatments to prevent cancer metastases. Therefore, it is imperative to provide more effective biomarkers and targets for the diagnosis and treatment of cancer metastases. The metastasis process of tumor cell consists of a series of cell biological events, including the primary tumor cells gaining the ability of invasion and migration, entering and spreading within the circulation system, exudating and migrating to the distant organs, and finally proliferating in the novel loci to form metastases. In the circulation system, most of the detached tumor cells undergo programmed cell death, but a tiny fraction obtains anoikis resistance which is critical and prerequisite for metastasis. The solid tumor cells are surrounded by a complex microenvironment which challenges the drug delivery and immune cell performance. In the circulation system, the absence of extracellular matrix makes it much easier to fight tumor cells. Many strategies have been proposed to inhibit the metastasis of tumor cells by accelerating their anoikis.

Autophagy is a unique and conservative material degradation and recycling process in eukaryotes which is important for cell survival in stressful environments. It's reported that enhanced autophagy facilitates anoikis resistance of many tumor cells, such as glioma, prostate adenocarcinoma, hepatic cancer. Meanwhile, inhibiting autophagy can promote anoikis and suppress metastasis of these cancers, which suggests that it's a potential strategy to avoid tumor metastasis by targeting autophagy. Autophagy process is mediated by a series of ATGs (Autophagy related genes). Among them, ATG4B, a cysteine protease, carries out the lipidation and delipidation of MAP1LC3/LC3 which is essential for phagophore expansion and autophagosome maturation. Our group has clarified many mechanisms about the expression, subcellular localization, posttranslational modification and activity of ATG4B in different tumors. Recently, we published our latest research in the journal AUTOPHAGY about promoting the anoikis of gastric cancer (GC) cells by enhancing the ubiquitin-dependent degradation of ATG4B.

In our recent research, we found that ATG4B was upregulated in detached GC cells compared with adherent ones, and ATG4B could enhance anoikis resistance in GC cells. It indicated that ATG4B could be a potential target to reduce the anti-anoikis and metastasis capacity of GC. Targeting ATG4B has been reported to inhibit tumor proliferation and metastasis significantly *in vitro*. Research indicated that ATG4B deficiency mice are viable and fertile, but these mice are susceptible to many diseases, including diabetes, muscle atrophy and myocardial damage which suggests that targeting ATG4B directly is not a satisfactory choice. Hence, clarifying the detailed mechanism of ATG4B upregulation is of great significance to inhibit its function. In view of this consideration, our further research has made some gratifying findings.

In addition to binding with proteins, RBP2GO analysis displayed that ATG4B also has the potential to bind with non-polyA tailed RNA, including snRNA, rRNA, tRNA, lncRNA, circRNA and so on. CircRNAs have many advantages as biomarker for diagnosis and treatment of diseases. In our research, we identified many potential ATG4B-interacting circRNAs by RIP-seq, among which *circ*SPECC1 could inhibit the expression of ATG4B. Consequently, we confirmed the interaction of ATG4B with *circ*SPECC1 by RIP-qPCR and RNA pull down. What's more, the binding domains both in ATG4B and *circ*SPECC1 were identified by truncating and site-specific mutation. Further, we found that *circ*SPECC1 downregulated the ATG4B protein by enhancing its ubiquitin-dependent degradation. Mechanically, *circ*SPECC1, acting as a scaffold, promoted the interaction of ATG4B with its E3 ubiquitin ligase RNF5. Of great interest, ATG4B can undergo liquid-liquid phase separation (LLPS) in GC cells. We identified the intrinsically disorder regions (IDRs) in the ATG4B protein which is responsible for its LLPS. Additionally, we revealed that *circ*SPECC1 promoted the LLPS of ATG4B (**Figure 1**). However, *circ*SPECC1 is downregulated in GC which is in line with the high expression of ATG4B. Therefore, restoring the *circ*SPECC1 abundance in GC cells could be a potential strategy to inhibit ATG4B-induced GC metastasis.

**Figure 1.**
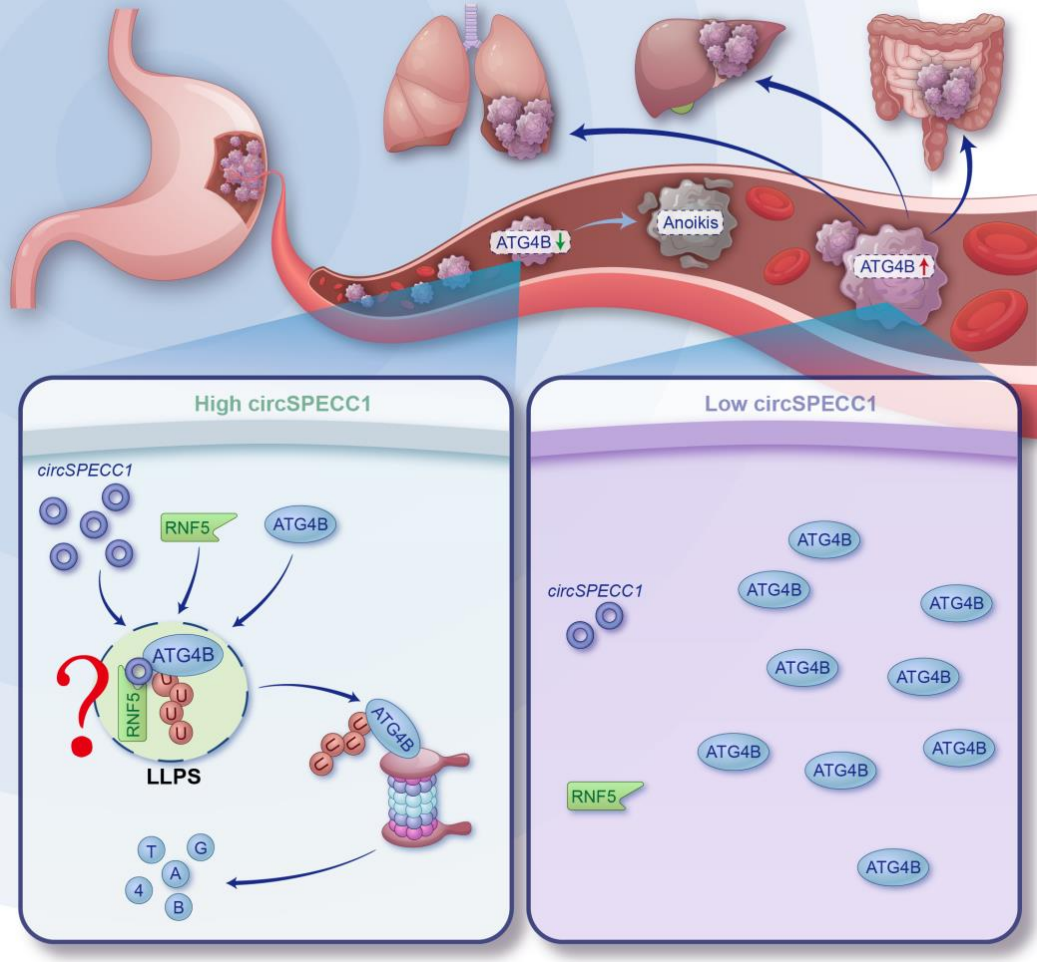
FIGURE 1: C*irc*SPECC1 promotes the LLPS and ubiquitination of ATG4B. Metastasis is the most fatal threat of cancer patients. Among the multitudinous mechanisms, enhanced autophagic progress is a critical aspect. As the key participant of autophagy, ATG4B promotes gastric cancer cells metastasis by increasing anoikis resistance. The *circ*RNA *circ*SPECC1 can promote the interaction of ATG4B with its E3 ligase RNF5. What's more, *circ*RNA can facilitate the LLPS of ATG4B and may thereby provide an optimal reaction environment for RNF5-mediated ubiquitination of ATG4B; however, more direct evidences are required to confirm this. Additionally, detailed functions and mechanisms of ATG4B in the LLPS puncta are also unclear.

Our specific study indicated that *circ*SPECC1 was hypermethylated in N6-adenylate (m^6^A) which can be recognized by m^6^A reader to determine its stability. Accordingly, we screened the potential m^6^A reader that recognize *circ*SPECC1 by online bioinformatic tools and confirmed that ELAVL1 could bind with *circ*SPECC1 and inhibit its expression in a m^6^A-dependent manner. Therefore, we suppose that blocking the interaction of ELAVL1 with *circ*SPECC1 could restore *circ*SPECC1 expression. Fortunately, we screened out an FDA-approved compound, lopinavir, which can inhibit the interaction of *circ*SPECC1 and ELAVL1. The further research showed that lopinavir treatment increased *circ*SPECC1 abundance, inhibited ATG4B expression, promoted anoikis and reduced metastasis of GC cells both *in vitro* and *in vivo*.

Of course, our research is not perfect. We believe that at least five aspects of our study need further investigation. First, the occurrence of ATG4B LLPS is a non-physiological condition, and further investigation in physiological situation is needed. But to be honest, this is a common shortcoming of many LLPS researches. Meanwhile, more function and mechanism of ATG4B in the LLPS puncta is also worthy of study (**Figure 1**). Second, the direct evidence about the relationship between LLPS and ubiquitination of ATG4B are also insufficient. Third, it's of great significance to clarify the m^6^A writer and eraser that modify *circ*SPECC1. Fourth, tumor metastasis involves multiple processes, whether lopinavir impacts other processes of metastasis is unclear. Finally, the clinical investigation to confirm the anti-metastasis capacity of lopinavir is lacking. However, the flaws do not detract from the jade's essential beauty. To the best of our knowledge, this study presented the first evidence that ATG4B was a novel RBP to bind with *circ*RNAs and can undergo LLPS. Binding with *circ*RNA can regulate the LLPS and ubiquitination of ATG4B. Besides, we proposed a novel strategy to inhibit ATG4B and ATG4B-induced cancer progression. Additionally, we screen out lopinavir, an FDA-approved compound which indicates higher safety and clinical application prospect, to inhibit the metastasis of GC.

In conclusion, our study demonstrated for the first time that the ELAVL1-*circ*SPECC1-ATG4B-autophagy signaling pathway is critical for the survival of cells under detached conditions, corresponding to nodes that the tumor cells are spreading in the circulatory system. Meanwhile, screened out lopinavir to suppress this pathway and inhibit GC metastasis. All of our researches are to identify novel therapeutic targets and strategies to inhibit cancer metastasis.

